# Inequalities, barriers, intersectionality, and facilitators of careers of women with disabilities: Themes and future research agenda from a scoping review

**DOI:** 10.3389/fpsyg.2023.1104784

**Published:** 2023-10-25

**Authors:** Xi Wen Chan, Kate Hutchings

**Affiliations:** ^1^Centre for Work, Organisation and Wellbeing, Griffith University, Brisbane, QLD, Australia; ^2^Department of Employment Relations and Human Resources, Griffith University, Brisbane, QLD, Australia

**Keywords:** career, development, disabilities, motivations, opportunities, scoping review, women

## Abstract

This article examines the career motivations, opportunities, and development of women with disabilities. The increasing number of women in paid work over recent decades has resulted in substantive research on all aspects of women’s careers, yet examination of women in some minority groups has received much less attention. Extant research has found that although people with disabilities exhibit strong organisational loyalty and less absenteeism from work, they experience a disability pay gap, and are less likely to be working as managers or directors or in professional occupations. Experiences of prejudice, ableism, and discrimination lead to fear of disclosure, and this may be accentuated in some economies and communities where there is stigma associated with having disabilities, especially mental illness. As estimates suggest that one billion people in the world have a disability, and women may experience a double disadvantage based on their gender and disability and thus have lower levels of employment than men with disabilities and women without disabilities, it is important to understand factors affecting the career motivations, opportunities, and development of this significant proportion of the (potential) workforce. This article presents a rapid review of the relevant literature and identifies key themes including career inequalities for women with disabilities, career barriers for women with disabilities, educational interventions to improve career motivations and opportunities for women with disabilities, facilitators for careers and career development of women with disabilities, and intersectionality of gender, disability, and other identities for women’s career development. Limitations of the review include the exclusion of grey literature and certain databases in the search process. Based on the analysis of the themes developed from peer reviewed academic literature examined, suggestions for organisations and governments are presented, and a future research agenda established.

## 1. Introduction

This article examines the career motivations, opportunities, and development of women with disabilities internationally including studies undertaken in advanced, emerging, and developing economies. As recent decades have evidenced increasing numbers of women in paid employment throughout the world, there has been significant research examining aspects of women’s work (Flores et al., 2021). Studies conducted on women’s careers have examined gender pay equity (Bishu and Alkadry, 2017), women’s representation in management and leadership roles (Lyness and Grotto, 2018; Morgenroth et al., 2020), women’s work/life balance (Singh et al., 2022) and work/life conflict (Gisler et al., 2018) (and associated spillover, crossover, or boundaries between work and life) (Bakker et al., 2009; Kelliher et al., 2019). Similarly, substantive research has been undertaken on women’s career development (Phillips and Imhoff, 1997), women’ career transitions (Greer and Kirk, 2022), career mentoring for women (Ehrich, 2008), and women’s career motivations (Allen et al., 2016). However, as limited research has been conducted on the career motivations, opportunities, and development of women with disabilities, this article seeks to provide insights into the experiences of this significant but neglected part of the workforce internationally.

Disability includes physical, sensory, intellectual, head or brain injury, psycho-social (mental illness/mental health issues), and other conditions restricting everyday activities, which may be permanent or temporary (Australian Public Service Commission [APSC], 2022), and both visible and invisible. Extant research has found that although people with disabilities have less absenteeism from work, strong organisational loyalty, and high retention rates (Australian Network on Disability, 2023), they experience a disability pay gap, are less likely to be working as managers or directors or in professional occupations than working people who do not have, or identify as having, a disability. Moreover, their experiences of prejudice, ableism, and discrimination also lead to a fear of “coming out” and many who have disclosed would not do so again (Marshall et al., 2020). Estimates suggest that one billion people in the world have a disability and numbers are comparable across men and women (Schur et al., 2016). However, women with disabilities have lower rates of employment, are less likely to participate in education or training, and are more likely to live in poverty than men with disabilities (World Health Organization (WHO), 2011, cited in Lindstrom et al., 2020b). The proportion of society with a disability is also increasing given ageing populations and disability generally increases with age. People are living (and working) longer than in previous generations (Gratton and Scott, 2016), thus more women with disabilities will be seeking to work and pursue careers.

Across advanced, emerging, and developing economies internationally, restrictive gender roles and low expectations of people based on their disability/ies lead to lack of opportunities in the workforce (Noonan et al., 2004, cited in Lindstrom et al., 2020b). The United Nations Sustainable Development Goals (SDGs) include SDG 5 Gender Equality, SDG 8 Decent Work and Economic Growth, and SDG 10 Reduced Inequalities (United Nations [UN], 2022). Improving women’s engagement with, and progression in, their careers is central to advancing these three SDGs. However, women with disabilities may experience a double disadvantage in their careers given their intersecting gender and disability/ies, particularly in countries or communities in which there is stigma about having a disability (e.g., Parnes et al., 2009; McConkey et al., 2016). Although there have been investments in education and women’s development programs to improve labour force participation and career progression of women by national governments in advanced economies and some developing and emerging economies (e.g., Patrinos and Psacharopoulos, 2010), the Global Gender Gap Index highlights women in developing economies fare especially poorly relative to developed economies (World Economic Forum [WEF], 2022) and women of minority groups experience a greater gender gap. Central to improving the position of women with disabilities in workplaces is understanding their career motivations and development, and how these are affected by a range of barriers and facilitators within organisations and societies.

This research involved a rapid review of literature to examine hems and determinants emerging from the analysis of inequalities, barriers, intersectionality (overlapping aspects of an individual’s identity, including, but not limited to ethnicity/race, gender, and sexuality), and facilitators of the career motivations, opportunities, and development of women with disabilities. Given the need to scope the current literature in a timely manner to set an agenda for more research in this important area, this research used a rapid literature review approach (Tricco et al., 2017). This approach provides an accelerated but simplified form of systematic review involving: development of research question/s; defined inclusion and exclusion criteria; searches of databases (i.e., Google Scholar, ProQuest, and Scopus); extraction of data from the selected studies; analysis and synthesis off the findings of the studies; and write-up of the findings into key themes. The research was initiated with the intention to broadly understand women with disabilities and their career experiences in respect to inequalities relative to others, and barriers and facilitators/strategies to enhance career outcomes (which is a starting point for much research on aspects of work and careers of people in minority groups). The research questions were then refined and expanded following the literature search and analysis of key themes from the literature. That is, in addition to barriers and facilitators, it became evident that educational interventions were a significant component of the research in examining implications for facilitating careers of women with disabilities, and thus was introduced as a separate research question. Further, though in commencing the research it was evident that women with disabilities suffer disadvantage from both of these aspects of their identities (gender and disability), which could be considered as part of career inequalities, within the publications there was a stream of discussion around other forms of intersectionality such as ethnicity. Thus, intersectionality was also added as a separate research question. Specifically, the following research questions are addressed:

RQ1: What are the career inequalities for women with disabilities (relative to men with disabilities)?

RQ2: Which barriers affect careers for women with disabilities?

RQ3: What educational/curriculum interventions improve career motivations and career opportunities for women with disabilities?

RQ4: What facilitators/strategies are there for careers/career development for women with disabilities?

RQ5: How does intersectionality affect the career development of women with disabilities?

The next section of the article presents the review of literature including the method, findings, and themes. The article then provides a discussion of the key findings, strengths and weaknesses of the literature, implications for governments and organisations, and a future research agenda. The article concludes with limitations of the review and key contributions.

## 2. Review of literature

### 2.1. Method–protocol and search strategy

The rapid scoping review was conducted based on protocol guidance from Tricco et al. (2017). Given the rapid, exploratory nature of this review, the scholarly publications were sourced from three main databases–namely, Google Scholar, Scopus, and ProQuest. The search strategy used these key terms and boolean operators [(women with disability* OR female employee* with disability* OR female* with disability* OR disabled women OR disabled female employee*) AND (career*)]. In this article the authors use the term women (being a gender identity) as distinct from female (which is biological sex assigned at birth). However, the term female was used in the searches to ensure that all relevant publications were found. The searches were not limited to any academic disciplines, specific areas of employment/work, or geographical regions.

#### 2.1.1. Inclusion and exclusion criteria

The searches were, however, limited to peer-reviewed journal articles, book chapters, and dissertations published or made available online until September 2022. Other sources (e.g., industry reports, commentaries, and datasets) were not included in the search, since they are typically considered grey literature and it is difficult to ascertain if they have been peer-reviewed.

#### 2.1.2. Screening process

**FIGURE 1:**
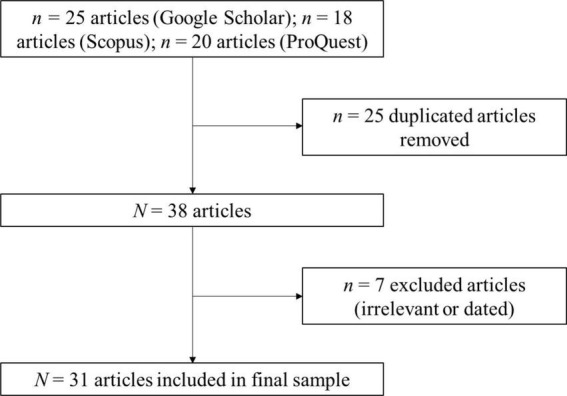
Screening process flow diagram.

To capture as many relevant publications as possible, the researchers undertook a backward and forward citation search of the publications already identified. The initial search process yielded 25 publications in Google Scholar, 18 publications in Scopus, and 20 publications in ProQuest. The researchers then combined and removed duplicated articles across the three search platforms, yielding an initial list of 38 publications. Each researcher then read the publications independently to determine if they focused on women with disabilities and their career-related motivations and/or development, and whether they should be included in this literature review. Each researcher then consulted with the other researcher to determine the final sample of publications on which this review is based, and the consultation process resulted in a total of seven publications being removed. Of these, three did not involve research in relation to the topic or context, and the other four were dated publications which the authors could not access. Therefore, the final sample consisted of 31 publications (see Figure 1).

#### 2.1.3. Characteristics of the studies

Most of the publications (*n* = 23) were empirical studies. Nine were quantitative studies, 10 were qualitative studies, four were case studies, four were conceptual publications, three publications were editorial articles, and the remaining publication was a policy review article (see Table 1). The studies were published between 1996 and 2022. Most of the publications were in the careers discipline, and a handful were published in fields such as equity, diversity and inclusion, education, gender, disability, rehabilitation, and psychology. The authors met over the course of a few weeks to go through each of the 31 scholarly publications to identify common topics and themes examined in relation to the inequalities, barriers, intersectionality, and facilitators of the career motivations, opportunities, and development of women with disabilities.

**TABLE 1:** Included publications (31 in total).

References	Title	Year	Source	Research design	Sample (for empirical publications only)	Type of disability/ies
Omolawal and Obisesan, 2022	Social support: Implications for career optimism and achievement among women and girls with disabilities in Ibadan, Nigeria	2022	Gender and Behaviour	Qualitative	Interviews with 24 women aged over 18 with disabilities and 5 family members/support people	Intellectual, Physical–unable to walk, Sensory–deaf, blind, vision impaired, dumb (unable to speak)
Scott, 2022	A trifecta?: Exploring the intersection of eace, gender, and disability on the career advancement efforts of college-educated African American women with physical disabilities	2022	Creighton University ProQuest Dissertations Publishing	Qualitative	Interviews with eight college-educated African American women with physical disabilities aged over 25	Muscular Dystrophy, Cerebral Palsy, amputee, Ramsey Hunt Syndrome, Multiple Sclerosis, Epilepsy, Deafness
Hirano et al., 2022	Examining the role of peer support on work experiences for young women with disabilities	2022	Journal of Career Development	Quantitative	Survey of 366 girls in years 9–12 including girls with various disabilities and a control group without disabilities	Learning, Other health impairments, Emotional disturbance [Psycho-social], Intellectual, speech or language impairment
Hanlon and Taylor, 2022	Workplace experiences of women with disability in sport organizations	2022	Frontiers in Sports and Active Living	Qualitative	8 interviews with women with physical or sensory disabilities in paid or voluntary roles in sports organisations	Physical – physical impairment Sensory – vision impairment, hearing impairment
Jungwirth and Bakhshizadeh, 2022	Wiedereinstieg mit Hindernissen–die Teilhabe von Frauen* mit Behinderung oder chronischer Erkrankung an Arbeit	2022	GENDER–Zeitschrift für Geschlecht, Kultur und Gesellschaft	Qualitative	Interviews in counselling centre – number not available	Disabilities, chronic disease
Walden et al., 2021	Paths 2 the future college and career readiness curriculum: Recommendations for school psychologists	2021	Journal of Applied School Psychology	Conceptual	N/A	N/A
Pham et al., 2020	Future aspirations of young women with disabilities: An examination of social cognitive career theory	2020	Career Development and Transition for Exceptional Individuals	Quantitative	Survey of 366 girls/young women with various disabilities in school years 9–12	Mental health [Psycho-social] including depression and anxiety, diabetes, Learning, Emotional behavioural disorder (Psycho-social), Intellectual, speech language impairment, Autism Spectrum Disorder, multiple dis-abilities, hard of hearing, visually impaired, traumatic brain injury, orthopaedic impairment
Lindstrom et al., 2020a	Paths 2 the future: Evidence for the efficacy of a career development intervention for young women with disabilities	2020	Exceptional Children	Quantitative	Survey of 366 girls/young women with various disabilities in school years 9–12	Mental health [Psycho-social] including depression and anxiety, diabetes, Learning, Emotional behavioural disorder (Psycho-social), Intellectual, speech language impairment, Autism Spectrum Disorder, multiple dis-abilities, hard of hearing, visually impaired, traumatic brain injury, orthopaedic impairment
Lindstrom et al., 2020b	Finding our voices: Employment and career development for women with disabilities	2020	The Palgrave Handbook of Disability at Work	Conceptual	N/A	N/A
Ballo, 2020	Labour market participation for young people with disabilities: The impact of gender and higher education	2020	Work, Employment and Society	Quantitative	From data set of 20,207 people aged 20–35 years receiving a disability benefit	Not specified
Lindstrom et al., 2019	“Learning to Be Myself”: Paths 2 the future career development curriculum for young women with disabilities	2019	Journal of Career Development	Quantitative	Survey of 49 (41 for second survey) of girls aged 14–20 (years 9–12) with various disabilities. Focus groups with 33 from the first sample for the focus groups	Learning, Health [Physical], Intellectual, Emotional [Psycho-social]
Heydemann and Johnson, 2019	Eradicating wealth inequality includes achieving equal pay	2019	Contexts	Policy	N/A	N/A
Peter et al., 2018	Reassessing cultural capital: access to employment for women with disabilities in Saudi Arabia	2018	Equality, Diversity and Inclusion	Qualitative	Interviews with 9 women with physical or sensory disabilities	Physical–not specified, Sensory–visual disability, hearing disability
Valtonen, 2017	Woman of her own life–Improving the career management skills of women with disabilities	2017	Journal of Mental Health Research in Intellectual Disabilities	Conceptual	N/A	N/A
Hampton et al., 2015	The influence of family of origin on the career development of outstanding women with disabilities in China	2015	Journal of Rehabilitation	Qualitative	Interviews with 14 women with (various) disabilities aged 18–60 who had received Outstanding Person with Disabilities Award	Physical, Sensory – Blind, low vision, Deaf, Multiple disabilities
Conyers et al., 2014	Addressing the career-related needs of women with disabilities	2014	Career Development, Employment, and Disability in Rehabilitation: From Theory to Practice	Conceptual	N/A	N/A
Lindstrom et al., 2004	Expanding career options for young women with learning disabilities	2014	Career Development and Transition for Exceptional Individuals	Case Study	Six interviews with employed and unemployed young women with learning disabilities (with two having other disabilities also) 28 interviews with key informants (e.g., parents, transition specialist, rehabilitation counsellor)	Learning–one with secondary congenital spinal disability, and one with secondary emotional disturbance [Psycho-social]
Lindstrom et al., 2013	Building career PATHS (post-school achievement through higher skills) for young women with disabilities	2013	The Career Development Quarterly	Quantitative	110 surveys from girls aged 14–21 (years 9–12) with various disabilities. 68 focus group participants (from those surveyed)	Learning, Autism Spectrum Disorder, multiple disabilities, Intellectual, Other health impairment, visual impairment, hearing impairment, orthopaedic impairment
Lindstrom et al., 2012	Gender gaps: Career development for young women with disabilities	2012	Career Development and Transition for Exceptional Individuals	Qualitative	Five focus groups with 34 high school and college young women with various disabilities. 26 interviews with employers, special education teachers and school administrators	Learning, Attention Deficit Disorder/Attention Deficit Hyperactivity Disorder, Autism, Multiple disabilities, Mental retardation [Intellectual], Speech/language
Miesch, 2011	An investigation of background and contextual variables related to career decision self-efficacy and vocational outcome expectations for college women with learning disabilities	2011	University of Oregon ProQuest Dissertations Publishing	Quantitative	Survey of 136 undergraduate student women with learning disabilities	Learning (and with secondary disability)–Attention Deficit Hyperactivity Disorder, mental health [Psycho-social], traumatic brain injury
Wehmeyer et al., 2009	Promoting self-determination and self-directed employment planning for young women with disabilities	2009	Journal of Social Work in Disability and Rehabilitation	Conceptual	Brief reference to some preliminary data	N/A
Lindstrom et al., 2008	Building opportunities for young women with disabilities	2008	Teaching Exceptional Children	Qualitative	Curriculum development intervention with 102 young women with various disabilities aged 16–20 (years 10–12)	Learning, Emotional [Psycho-social], orthopaedic impairment, mental retardation [Intellectual], hearing impairment, vision impairment, other health impairment, traumatic brain injury
Noonan et al., 2004	Challenge and success: A qualitative study of the career development of highly achieving women with physical and sensory disabilities	2004	Journal of Counselling Psychology	Qualitative	Interviews with 17 high-achieving employed women with physical and sensory disabilities	Physical–spinal cord injury, Post-Polio Syndrome, mobility impairments, Rheumatoid Arthritis, Sensory - Blind, Deaf
Haq, 2003	Career and employment opportunities for women with disabilities in Malaysia	2003	Asia Pacific Disability Rehabilitation Journal	Qualitative	Interviews with 3 employed professional women with physical disabilities	Physical–spinal cord injury, Polio
Lindstrom and Benz, 2002	Phases of career development: Case studies of young women with learning disabilities	2002	Exceptional Children	Case Study	Case studies of six employed and unemployed young women with disabilities. 34 interviews with young women with learning disabilities (and three having other disabilities),	Learning–one with secondary congenital spinal disability, and one with secondary Emotional disturbance [Psycho-social], and one with secondary depression
Reed, 2002	Self-concept and social isolation in career development: A study of the dual disadvantage of women with disabilities	2002	University of Arkansas ProQuest Dissertations Publishing	Quantitative	57 women 31 with disabilities and 26 without) completed a 20-h workshop and pre-and post-tests. Of the 57, 39 women completed interviews 20 with disabilities and 19 without disabilities	Physical, Sensory, chronic illness, Emotional [Psycho-social], Learning
Keim et al., 2002	Examining the differences in career thoughts of women in three low socioeconomic status groups	2002	Journal of Employment Counselling	Quantitative	Career Thoughts Inventory with 25 women with disabilities (receiving job placement services from a state-funded, community-based programme)	Not specified
Lindstrom, 2000	Patterns of career development: Case studies of young women with disabilities entering the workforce	2000	University of Oregon ProQuest Dissertations Publishing	Case Study	Case studies of six employed and unemployed young women with disabilities. 34 interviews with young women with learning disabilities (and three having other disabilities), their parents, school and vocational rehabilitation personnel, and their employers	Learning–one with secondary congenital spinal disability, and one with secondary emotional disturbance [Psycho-social], and one with secondary depression
Reed, 1999	Women with disabilities making the transition back to work: Psychosocial barriers and interventions	1999	Work	Editorial	N/A	
Runte, 1998	Women with disabilities: Alone on the playground	1998	Canadian Woman Studies	Individual self-reflection	Physical disability from vascular disease	
Nosek and Bennett, 1996	Career development of women with physical disabilities	1996	Journal of Rehabilitation Research and Development	Qualitative	Interviews with 11 employed and unemployed women with physical disabilities	Physical–amputation, spinal cord injury, Polio, Spina Bifida, Cerebral Palsy, Muscular Dystrophy, Multiple Sclerosis

In respect to type of disability/ies, the categorisation is as provided by the author/s. Where specific disabilities, diseases, or impairments are mentioned by the author/s, these are also included. It should be noted that the terminology is as described by the author/s and some of this terminology is not in current usage. Table 1 are the authors of this articles’ reference to current terminology.

Table 1 presents an overview of the research design (conceptual, quantitative, qualitative, and mixed) and sample of each of the 31 articles. Some of the publications referred to their study being of women with disabilities in general, whilst others specified particular disabilities of the women researched. Table 1 also contains a column titled “Type of Disability,” under which the categorisation provided by the author(s) is listed. Where specific disabilities, diseases, or impairments are mentioned by the author(s), these are also listed. It should be noted that the terminology is as described by the author(s) and some of this terminology is no longer in common usage. Utilising the definition from the Australian Bureau of Statistics’ Survey of Disability, Ageing and Carers including “Physical,” “Sensory,” “Intellectual (including learning),” “Head injury, stroke, or acquired brain injury,” “Other–long-term conditions restricting everyday activities,” and “Psycho-social (mental health and illnesses)” (Australian Public Service Commission [APSC], 2022), the type of disability mentioned by the author(s) for some of the publications is referred to with these categories and shown in Table 1. A wide range of disabilities were examined in the research including physical, sensory, and learning, with a small number of publications including samples of women with psycho-social disabilities. In some instances, the publications focused on primary learning disabilities but also mentioned other secondary disabilities.

## 3. Findings

**FIGURE 2:**
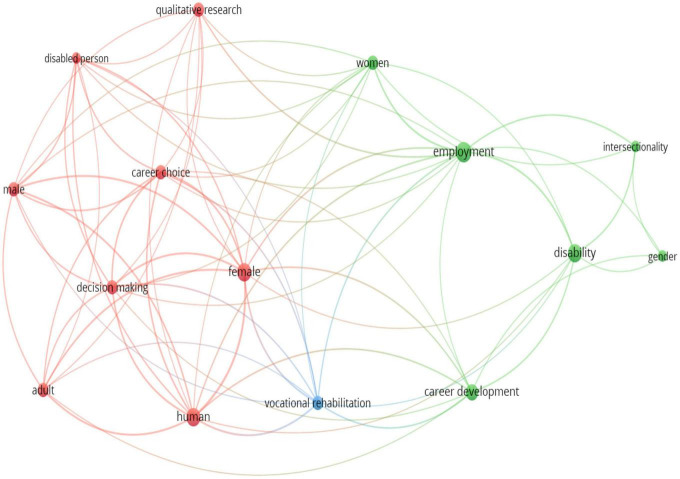
Bibliometric map of the research articles’ keywords using VOSviewer.

Figure 2 presents a bibliometric map^1^ of the key topics explored in the 31 scholarly publications. The size of each word represents the frequency of the word being mentioned. The distance between two words indicates their correlation: shorter distances represent stronger correlations. Three clear clusters emerged regarding the inequalities, barriers, intersectionality, and facilitators of the career motivations, opportunities, and development of women with disabilities. The largest cluster is the red cluster, represented by the words “female,” “decision-making,” “career choice,” “human,” “adult,” “male,” “disabled person,” and “qualitative research.” The next cluster is the green cluster, represented by the words “women,” “employment,” “career development,” “disability,” “intersectionality,” and “gender.” The independent blue cluster of “vocational rehabilitation” also emerged and was related to both the red and green clusters. These three clusters provide a high-level overview of the five recurrent themes that emerged from the 31 publications, which are discussed in detail in the next section.

Several recurring themes emerged while the researchers read and reviewed each publication more closely, guided by the key words and topics that emerged in the bibliometric map. Specifically, the themes identified were: Theme 1 – Career inequalities for women (vs. men) with disabilities (Reed, 2002; see Keim et al., 2002; Heydemann and Johnson, 2019; Ballo, 2020), which falls under the red cluster in Figure 2; Theme 2–Barriers to careers for women with disabilities (see Nosek and Bennett, 1996; Runte, 1998; Reed, 1999; Haq, 2003; Noonan et al., 2004; Conyers et al., 2014; Peter et al., 2018; Hanlon and Taylor, 2022; Scott, 2022), which falls under the green cluster in Figure 2; Theme 3–Educational/curriculum intervention to improve career motivations and opportunities for women with disabilities (see Lindstrom, 2000; Lindstrom and Benz, 2002; Lindstrom et al., 2008, 2012, 2013, 2004, 2019; Wehmeyer et al., 2009; Miesch, 2011; Pham et al., 2020; Walden et al., 2021; Hirano et al., 2022), which falls under the green cluster in Figure 2; Theme 4–Facilitators/strategies for careers/career development of women with disabilities (see Nosek and Bennett, 1996; Runte, 1998; Reed, 1999; Keim et al., 2002; Haq, 2003; Noonan et al., 2004; Conyers et al., 2014; Hampton et al., 2015; Valtonen, 2017; Hanlon and Taylor, 2022; Scott, 2022), which falls under the green cluster in Figure 2; and Theme 5–Intersectionality of gender, disability, ethnicity, and low socio-economic status (amongst others), and its effects on career development (see Keim et al., 2002; Haq, 2003; Noonan et al., 2004; Ballo, 2020; Lindstrom et al., 2020b; Hanlon and Taylor, 2022; Scott, 2022), which falls under the green cluster in Figure 2. Each of these themes, which address each of the research questions, along with the bibliometric clusters will be explored and discussed in detail in the following paragraphs.

### 3.1. Theme 1–Career inequalities for women (vs. men) with disabilities

A few studies focused on the career inequalities of women with disabilities in a specific country (e.g., Norway, US), typically using a comparison group of men with disabilities in the same country (Heydemann and Johnson, 2019; Ballo, 2020), and examining issues (e.g., education, labour market participation, wages) closely related to the careers of women with disabilities. This theme is represented by the keywords “female,” “male,” “career choice,” “decision making,” and “disabled person” in the red cluster of Figure 2. Studies that are under this theme have also tended to examine women with disabilities broadly, without specifying the types of disability.

Consistent with findings on the gender pay gap, a significant wage gap also exists for women with disabilities who are employed as compared to men with disabilities who are employed (Keim et al., 2002; Heydemann and Johnson, 2019). However, the studies’ findings do not converge on women with disabilities as being significantly more disadvantaged than men. As an example, based on a sample of 20,207 disability benefit recipients from the Norwegian full population register data, Ballo (2020) found that of those with higher education, 72% were women. More interestingly, analyses of the interplay between higher education and gender revealed that women with disabilities benefited more from higher education than men with disabilities, and higher education had a stronger effect on these women’s labour market success (Ballo, 2020). Reed (2002) also found no significant difference on measures of career self-concept (career decision-making self-efficacy, self-esteem, and assertiveness) and social isolation (role participation, role stress, and role conflict) for women with and without disabilities.

Based on these findings, it could be concluded that the inequalities and disadvantages that women with disabilities face are not prevalent. However, it is important to note that these studies did not consider any contextual (or macro) factors in the environment (e.g., national policies and programs) that may have influenced the findings. This presents an opportunity for scholars to examine the careers of women with disabilities through a multi-level, interdisciplinary lens that considers the national context or factors, which are often instrumental in dismantling the systemic career-related barriers faced by women with disabilities. Moreover, it is important to understand that career inequalities for women vary according to types of disability/ies (Scior et al., 2020). Physical and technological adjustments may be made by organisations for women who have physical, sensory or learning disabilities. However, consideration also needs to be given to the rights of women with disabilities to work and for organisations to provide pathways e.g., opportunities for career development for women with intellectual or psycho-social disability that address stigma and enhance inclusion in workplaces.

### 3.2. Theme 2–Barriers to careers for women with disabilities

It is well-established that women with disabilities face many barriers to their employment, which leads to limited work and career opportunities. This theme is represented by the keywords “employment,” “gender,” “disability,” “career development,” and “women” in the green cluster and the independent blue cluster of “vocational rehabilitation” in Figure 2.

Conyers et al. (2014) organised the barriers into four key domains - namely, medical/health conditions (e.g., health deterioration, pain, fatigue, decreased muscle strength, and medication side effects), financial/legal systems (e.g., disability legislation and rights, health insurance, housing subsidies, rehabilitation services, and criminal justice system), psycho-social factors (e.g., coping with discrimination, mental health issues, stigma associated with disability, gender and self-identity, domestic and sexual violence, and work meaningfulness), and vocational/occupational factors barriers (e.g., low levels of educational attainment, skills and job training, as well as gaps in work history). In certain industries dominated by men who do not have a disability, such as sports and manufacturing industries, a focus on ableism, defined as a “system of oppression that faces disabled people in our society, a system that marks disabled people as inferior and most importantly, other” (Liebowitz, 2018, p. 153), coupled with a masculine or men-dominated culture, continues to serve as an entrenched double disadvantage for women with disabilities (Runte, 1998; Hanlon and Taylor, 2022).

Unsupportive workplace practices (Nosek and Bennett, 1996) and managers (Hanlon and Taylor, 2022), such as a lack of family-friendly working conditions and a lack of understanding of disability and accommodation issues (Peter et al., 2018), also remain unaddressed in many workplaces. Such situations have led many women with disabilities to either reduce their work hours or leave their organisations, stalling their careers (Hanlon and Taylor, 2022).

In sum, the studies that examined the career barriers of women with disabilities all highlight the widespread, systemic barriers to employment of women with disabilities, which continue to hamper their career opportunities, development, and progression from entry-level to leadership positions. As with Theme 1, some studies under this theme did not specify the types of disability. Some that did mention type of disability include Hanlon and Taylor (2022) who specified that the eight women interviewed either had a vision, hearing, or physical impairment, and Nosek and Bennett (1996) who explained that the 11 women who participated in their research had a physical disability, such as an amputation, spinal cord injury, Polio, Spina Bifida, Cerebral Palsy, Muscular Dystrophy, or Multiple Sclerosis.

### 3.3. Theme 3–Educational/curriculum intervention to improve career motivations and opportunities for women with disabilities

Across the 31 studies reviewed, many either had tertiary educational attainment as a control or predictor of the employment or career outcomes of women with disabilities. However, educational attainment is also an educational outcome, which does not provide practical insights on how to improve the career motivations and opportunities for women with disabilities. Correspondingly, to address this gap, quite a few studies in this review (see Lindstrom, 2000; Lindstrom and Benz, 2002; Lindstrom et al., 2008, 2012, 2013, 2004, 2019, 2020a,b; Wehmeyer et al., 2009; Miesch, 2011; Pham et al., 2020; Walden et al., 2021; Hirano et al., 2022) examined educational or curriculum interventions that sought to enhance the career motivations and opportunities for *young* women with disabilities. This theme is represented by the keywords “employment,” “gender,” “disability,” “career development,” and “women” in the green cluster of Figure 2.

Most of these publications examined and assessed career development interventions aimed at enhancing young women’s career-related self-efficacy, specifically career decision self-efficacy (Miesch, 2011), underpinned by social cognitive career theory (Lent et al., 2002) or self-determination theory (Deci and Ryan, 2012). Both theories seek to account for human motivation in social contexts, driven by the interplay of individual and environmental factors. In the past decade, Lindstrom et al. (2013), Lindstrom et al. (2019), Lindstrom et al. (2020a), Walden et al. (2021) developed the PATHS (Post-school Achievement Through Higher Skills) curriculum and “Paths 2 the Future” (P2F) curriculum designed to meet the unique career development needs of young women with disabilities, and both curricula cover topics such as self-awareness, disability knowledge, gender identity, and career and college readiness. Pre- and post-quantitative and qualitative tests performed by the authors also revealed an increase in self-confidence, self-awareness, ability to identify strengths, knowledge of multiple career options, goal-setting, career planning, and personal empowerment. Given the curricula have not been examined by other groups of scholars, it is hard to determine their external validity or the extent to which the effectiveness of the curricula can be generalised to other groups or settings of women with disabilities.

Alongside educational or curriculum interventions, Hirano et al. (2022) also found perceived peer support to be a relatively malleable factor (which interventions can target) that promotes career self-efficacy and outcomes for young women with disabilities. Miesch (2011) also found that real-world work experiences, such as paid work, volunteer work, and internship experience, beyond educational or curriculum interventions, enhances the career decision self-efficacy and outcome expectations for college women with learning disabilities.

Studies under this theme were more transparent in reporting the types of disabilities of the young women. For example, they either had a learning disability, health impairment, emotional disturbance, intellectual disability, or speech or language impairment (Hirano et al., 2022); learning disability, emotional disturbance, or congenital spinal disability (Lindstrom, 2000; Lindstrom and Benz, 2002); learning disability, Attention-Deficit/Hyperactivity Disorder (ADD/ADHD), Autism, intellectual disability, or speech or language impairment (Lindstrom et al., 2012); learning disability (primary) and ADD/ADHD, emotional or mental health disability, physical disability, or traumatic brain injury (secondary) (Miesch, 2011); learning disability, health impairment, emotional behavioural disorders, intellectual disability, speech or language impairment, autism, hearing impairment, visual impairment, traumatic brain injury, or orthopaedic impairment (Pham et al., 2020). Although the types of disabilities were stated in many of these studies in describing the samples of young women with disabilities, most of these studies neither differentiated nor discussed the types of disabilities in respect to implications for the findings.

Together, the above findings suggest that educational or curriculum interventions, in combination with contextual factors such as social support and work experiences, increase the career motivations, advancement, and outcomes for women with disabilities.

### 3.4. Theme 4–Facilitators/strategies for careers/career development of women with disabilities

A few studies (see Nosek and Bennett, 1996; Runte, 1998; Reed, 1999; Noonan et al., 2004; Conyers et al., 2014; Scott, 2022) that examined barriers to careers (e.g., career opportunities, development, and advancement) for women with disabilities also examined the facilitators or strategies for their careers. Correspondingly, there were several overlaps between Theme 2 and Theme 4. This theme is represented by the keywords “employment,” “gender,” “disability,” “career development,” and “women” in the green cluster and the independent blue cluster of “vocational rehabilitation” in Figure 2.

First, similar to how they organised the barriers to careers of women with disabilities, Conyers et al. (2014) distinguished four types of facilitators–namely, medical/health conditions (e.g., job accommodations network, medical support for women’s health), financial/legal systems (e.g., social security, workplace planning and assistance, legal resources, employment discrimination protection), psycho-social factors (e.g., local women’s resource centre and disability network for emotional and mental health support), and vocational/occupational factors (e.g., vocational rehabilitation services, career exploration and research tools, and employment initiative for jobseekers). Beyond the structural enablers listed above, social support from family members, co-workers, and leaders (Noonan et al., 2004; Scott, 2022) was frequently mentioned as a key facilitator of career progression for women with disabilities. Valtonen (2017) also highlighted interventions (e.g., workshops) co-designed by experts and women with disabilities are effective in improving the career management skills of women with disabilities, although it should be noted that the workshop model has not been assessed by other scholars.

Although it is easy to conclude that stakeholders should focus on implementing more enablers and minimising the barriers to the careers of women with disabilities, both Haq (2003), Scott (2022) reminded us that not all women with disabilities are keen to advance their careers due to the complexities they face in finding a supportive workplace and manager. Some of their research participants were, in fact, content and satisfied with their jobs and positions and were not actively seeking promotion or further career advancement opportunities.

Similar to Themes 1 and 2, studies under this theme did not always specify the types of disability. In Noonan et al. (2004) study, the 17 women interviewed had physical (including Rheumatoid Arthritis) and sensory (including Blind, Deaf) disabilities, while the eight college-educated African American women interviewed in Scott’s (2022) study mainly had physical disabilities such as Multiple Sclerosis, Cerebral Palsy, and Muscular Dystrophy. In Haq’s study the women had Polio or spinal cord injury.

### 3.5. Theme 5–Intersectionality of gender, disability, ethnicity, and low socio-economic status (amongst others), and its effects on career development

Among the studies that drew on intersectionality theory (Collins and Chepp, 2013) to explore the careers of women with disabilities (see Keim et al., 2002; Haq, 2003; Noonan et al., 2004; Ballo, 2020; Lindstrom et al., 2020b; Hanlon and Taylor, 2022; Scott, 2022), it was insightful to note that many considered other aspects of a person’s identity such as their race, ethnicity, or cultural identity, education level, and socio-economic status, beyond gender and disability. Another positive aspect of these studies was that they also stated the types of disabilities being examined, including physical disability, emotional disability, sensory disability, vision impairment, amongst others. Collectively, the findings from these studies demonstrated that the experiences of woman with disabilities were varied. This theme is represented by the keywords “intersectionality,” “gender,” “disability,” “career development,” “women,” and “employment” in the green cluster of Figure 2.

Drawing on intersectionality theory typically leads to the assumption that demographic factors (e.g., race, gender, and disability) will intersect to affect the career progression and career development opportunities of women with disabilities (Collins and Chepp, 2013), but many of these studies (e.g., Noonan et al., 2004; Scott, 2022) showed that the factors did not always intersect. For example, a few of the 17 women interviewed by Noonan et al. (2004) did not regard gender as salient to their disability identities, and they identified more as a person with disability than as a woman. Nevertheless, what was consistent across these studies was that: (1) the women experienced some impact on their careers because of their disabilities; (2) positive self-belief (Noonan et al., 2004), self-image (Haq, 2003), self-concept (Scott, 2022), self-confidence and self-esteem (Hanlon and Taylor, 2022), and self-perception (Keim et al., 2002) contributed positively to their job satisfaction and career development, progression, and employment; and (3) higher educational attainment and social support for higher educational attainment opened up more and better career and employment opportunities for women with disabilities.

More than half of the studies under this theme did specify the types of disabilities, namely, Noonan et al. (2004), Lindstrom et al. (2020b), Hanlon and Taylor (2022), Scott (2022), and the types of disabilities ranged from physical and sensory disabilities to vision and hearing impairments.

## 4. Discussion

The research questions are addressed by drawing on an analysis of the themes developed in the findings to present strengths and weaknesses across the literature, organisational and government policy suggestions and areas for future research. The research questions included:

RQ1: What are the career inequalities for women with disabilities (relative to men with disabilities)?

RQ2: Which barriers affect careers for women with disabilities?

RQ3: What educational/curriculum interventions improve career motivations and career opportunities for women with disabilities?

RQ4: What facilitators/strategies are there for careers/career development for women with disabilities?

RQ5: How does intersectionality affect the career development of women with disabilities?

In response to RQ1, the review of the literature generally found that women with disabilities experienced more career inequalities than men with disabilities, suggesting a double disadvantage in the workplace of intersecting gender and disability identities. In response to RQ2, the literature highlighted a range of structural, societal, and organisational factors affecting the career opportunities and development of women with disabilities. The literature search and analysis also revealed a range of educational/curriculum interventions for young women with disabilities, and thus, in response to RQ3, this literature suggests the vital role that peer support and early interventions can play in affecting not only career motivations but also career outcomes for women with disabilities. In response to RQ4, there was a range of facilitators/strategies identified within the literature that can support the career development of women with disabilities which include addressing issues across a number of domains, namely, medical/health conditions, financial/legal systems, psycho-social factors, and vocational/occupational barriers, and providing interventions. Finally, with respect to RQ5, although there were diverse findings within the literature, it was generally suggested that intersecting identities affect the career development of women with disabilities.

Based on analysis of the themes and research design, countries of study, and theoretical underpinnings, herein the strengths and weaknesses within the limited extant research on careers, career development and career motivations of women with disabilities is discussed. Consideration is given to important implications of the research findings for government policy and organisations, and issues for future research.

### 4.1. Strengths of the literature

Whilst research on careers and career motivations generally is published in human resource management and organisational behaviour journals (including specialist career journals), a strength of this research area is that it has been published across a diversity of disciplines including education and health. This means that there is not only awareness of issues affecting the careers of women with disabilities across a range of areas which can feed into women’s career opportunities and outcomes, but there is also the potential for inter-disciplinary collaboration to address issues to facilitate their careers (e.g., secondary and tertiary education initiatives, and medical and counselling interventions).

A further strength within the research was that a number of publications highlighted intersectionality and the challenges faced by women who have a disability but may also have other intersecting identities besides gender and disability including ethnicity/race, which may compound the stigma and discrimination they experience in commencing and advancing their careers. Having knowledge of this intersectionality is important for both governments and organisations to address competing challenges experienced by women with disabilities and enable them to take concrete actions to effectively support women with disabilities as they enter and progress in their careers.

Based on the literature reviewed, top-down interventions initiated by managers and organisations (e.g., mentoring and networking, disability-inclusive policies and practices, accessible job application processes, and workplace accommodations) (Conyers et al., 2014) as well as peer-to-peer social support from family members and co-workers (Noonan et al., 2004; Scott, 2022) were particularly crucial in first building the skills and connections women with disabilities needed to advance in their careers, and creating a more inclusive and equitable workplace for women with disabilities.

### 4.2. Weaknesses within the literature

A number of the studies found in the literature search examined educational/curriculum intervention to improve career motivations and opportunities for women with disabilities. Whilst it is critical to understand factors that hinder and facilitate women entering into employment and developing careers, there were limited studies actually examining the experience of women as employees and factors affecting their career retention and advancement. As most of the studies were qualitative exploratory studies, it is appropriate that they have small sample sizes. Though two quantitative studies had survey respondents of 366 and more than 20,000, respectively, which are considered large quantitative sample sizes, the majority of studies were qualitative and included interviews and focus groups of between three and 68 participants. Given that approximately one in eight people in the world have some form of disability, these small sample sizes are a weakness within the literature reviewed and there needs to be much more insight provided through larger sample sizes. Thus, future research could privilege large scale quantitative studies or qualitative studies that have multiple approaches such as documentary/organisational policy text analysis to more fully capture experiences of employed women with disabilities, and policies and practices of employers and organisations supporting women employees with disabilities.

Most of the research was conducted in the USA and from the same team of researchers who examined interventions in high schools and produced a series of publications on these developments. Though there were studies conducted in Australia, Canada, China, Finland, Kingdom of Saudi Arabia, Malaysia, Nigeria, Norway, and one conceptual publication that referenced practices in a range of countries, and there were authors from the aforementioned countries as well as Zambia, the existing literature provides limited knowledge of the differences in career motivations and development of women with disabilities internationally. In addition, given the lack of findings in relation to the cultural context (for inductive studies) as well as the lack of measurement of cultural or contextual variables (for deductive studies), there was not enough evidence to discuss differences emerging from the various cultural contexts.

Further, a few articles were not specifically about women with disabilities but mentioned this as part of broader issues (e.g., gender pay gap). This suggests that there is insufficient focus on the specific issues and needs of women with disabilities. Another weakness across the publications was that some articles did not specify disabilities and how that affected careers, and other articles referred to physical disabilities and grouped them together with a range of disabilities under this heading. For instance, neurological diseases may be categorised separately as other long-term diseases, but they may have impacts on physical functioning in the form of mobility issues or effect sensory functioning such as vision. Therefore, the studies could have been more specific about the types of disabilities or inter-related impacts on functioning. It is also important to note that there can be differences in effect on functioning (and therefore performance) and required adjustments at work between genetic and/or developmental disabilities and acquired disabilities such as those arising from accidents. This is also important with respect to people who acquire a new disability whilst in their current employment, and hence may require adjustments and support that had not been previously provided to them (or others in the workplace) by their employer. Although such issues with terminology are reflective of the period of publication, it is important that correct terminology is used for research that is intended to inform organisations, government, and society about supporting careers of women with disabilities.

A further weakness within the literature is limited theoretical underpinning. Of the 31 publications, 15 did not have any theoretical motivation. The remaining publications used the following theories underpinning their analysis: career development theory, critical disability theory, cultural capital theory, grounded theory, intersectionality theory, self-determination theory, and social cognitive career theory. For this area of research to develop into the future and provide a foundation for further longitudinal, multinational studies, it is critical that it is anchored in theory for coherence and stronger contributions to the field.

### 4.3. Organisational suggestions

As noted, people with disabilities have strong organisational commitment and hence organisations that implement policies and practices to assist careers and career development of women with disabilities are not only ethical but experience business advantages. Supporting careers of women with disabilities to address career inequalities and barriers to entry and progression (Themes 1 and 2 findings) and facilitate career development (Theme 4 findings) may include a range of diversity awareness programs for reducing stigma and improving understanding of people with disabilities and the specific needs of women with disabilities. These practices could include inclusive language and accessible workspaces, employee assistance programs, flexible work practices adjusted to the needs of individual employees, reward practices that highlight the value of employees with disabilities, targeted training programs for career advancement/leadership, and mentoring women with disabilities.

### 4.4. Government policy suggestions

There have been substantive government initiatives in many developed countries (as well as emerging and developing economies) to provide awareness of, and support for, people with disabilities. However, more attention needs to be given to addressing the systemic barriers to entry and continued participation and career advancement of people with disabilities (noted in Themes 1 and 2 findings), such as focusing on the specific needs of women with disabilities who may also experience double or triple disadvantages from intersecting identities of disability, gender, ethnicity/race, sexuality, or age (noted in Theme 5 findings). At a national level, governments need to enact anti-discrimination legislation and laws that require specific adjustments to be made by organisations for people with disabilities. Advertising campaigns are also an important tool for raising awareness of the extent and form of disabilities and creating understanding to reduce stigmatisation and discrimination against people with disabilities. Further, government schemes to assist people with disabilities to move into work is also important (e.g., job finding services, apprenticeship/trainee schemes). Such social initiatives can have important flow-on effects to organisations who can then implement policies and practices to enhance the career opportunities and development of women with disabilities who may have multiple competing responsibilities to paid work and unpaid care.

Moreover, ensuring that women with all forms of disability have opportunities for entry into, and inclusion within, workplaces as well as career progression necessitates organisational and governmental policies and structures for support across education, health, and social care (Scior et al., 2020).

### 4.5. Future research agenda

Although the literature review highlighted a number of important issues in relation to career motivations, opportunities and development of women with disabilities, there are a number of areas where further research is required.

First, much of the research reviewed examined women with physical disabilities or did not specify the type of disability of the research participants. More research is needed to examine the impact of specific disabilities on careers and, in particular, given that mental illness and mental health issues (also referred to as psycho-social disability) remain stigmatised in many countries and communities (e.g., Parnes et al., 2009; McConkey et al., 2016), it is important to examine the career opportunities of women with psycho-social disability across workplaces and countries. Moreover, more research should consider barriers and opportunities for both women with intellectual disabilities and/or learning disabilities to enhance their engagement and progression in careers.

Second, though some of the extant research has considered discrimination and stigmatisation of women with disabilities in workplaces, there is also a need for more research to specifically examine experiences across countries to consider how national cultural values in emerging and developing economies present different experiences as compared to developed countries that may already have established governmental and organisational infrastructure to support women with disabilities.

Third, though there is some research on intersecting identities of women with disabilities, future research could specifically consider the careers and career development of women with disabilities of other identifies (e.g., diverse sexualities), and comparisons of women in younger and older age groups in respect to their experiences including how motivations and opportunities have changed throughout varying life stages. Fourth, future studies could bring together research on work/life balance and work/family conflict and research on careers of women with disabilities to examine how organisations support women with disabilities to manage work/life balance including where the women may also have caring responsibilities for other family members with disabilities.

Fifth, though the extant research highlighted business organisations’ support (and, to a lesser extent, government support) provided to women with disabilities, future research could examine resources provided by international not-for-profit and non-governmental organisations to assist with career opportunities and development. Sixth, the extant research has tended to focus on women with disabilities working in business organisations, but future research also needs to examine careers for women working as contractors and in the gig economy and how this differs from more standard and stable employment. Seventh, though the limited extant research has examined women with disabilities in a range of countries, future research needs to include large-scale international studies of all aspects of work and careers for women with disabilities in more regions of the world (e.g., Africa, Asia, Latin America, the Middle East, Pacific Islands) that have received little attention in the literature, and which have less developed national and organisational policies for supporting people with disabilities. Moreover, research could examine best practices for supporting careers of women with disabilities within specific cultural contexts (and take account of socio-economic development within and across countries) and consider where such practices may be transferable to countries with similar socio-economic, cultural and policy contexts.

There are also some important issues that were not raised in the reviewed papers and themes in the findings that also warrant attention in future research. First, it is critical that employers/organisations have a broad understanding of the rights of women with disabilities. Though organisations internationally (and particularly in major, developed economies) have an increased focus on supporting diversity in organisations, they do not always implement legally mandated rights sufficiently (e.g., reasonable adjustment practices to accommodate needs of women employees with disabilities) and clearly provide knowledge about requirements to other employees throughout their organisation. Thus, it is important that future researchers analyse potential obstacles in organisations and provide guidelines to assist organisations to address barriers and ensure legal (and ethical) compliance.

Second, employers/organisations need better understanding of how to provide workplaces that are more accessible to improve opportunities for women with disabilities who may also have intersecting identities which necessitate other flexibility and adjustments. For instance, a woman with disabilities who also has caring responsibilities would benefit from workplaces providing individually tailored work hours that assist her to progress in her career whilst juggling eldercare, childcare, or her own medical appointments and other requirements. Future research might examine organisations that enact best practices in supporting women with disabilities with intersecting identities.

Third, it is important to understand attitudes of co-workers that can impede or facilitate career progression of women with disabilities. Even where organisations implement policies and practices to support people with disabilities, lack of uptake by co-workers can be an issue because they do not realise the barriers that affect people with disabilities from doing their work. In some instances, co-workers may even harass and bully colleagues with visible disabilities and women with disabilities may experience this more than other genders when they have intersecting identities that also experience discrimination such as older women, transgender women, or women of particular religious groups that are more visually identifiable than their men counterparts by their religious attire. Future research could examine the attitudes of co-workers and provide insights into how organisations educate their workers about supporting people with disabilities including raising awareness among employees and relevant stakeholders that physically and technologically accessible workplaces benefit all parties. This research could also draw parallels with research on bullying and harassment of minority groups in organisations to highlight the value of behavioural interventions and disability champions.

## 5. Conclusion

### 5.1. Limitations of the review

Of the 31 publications, four were dissertations, three were book chapters, and the remainder were journal articles. Though the intention was to focus on academic research, a limitation of the review was that grey literature was not examined. Future reviews might also examine this literature in English and other languages (e.g., government reports, industry reports, websites or reports from international organisations/peak bodies for people with disabilities) to provide further insights into the issues identified in this review. In addition, the accelerated nature of the rapid review may have resulted in a less comprehensive search as some studies in other databases may not have been captured. Therefore, the conclusions from this review should be interpreted with caution.

### 5.2. Contributions and summary

This article presents a rapid review of the existing literature on women with disabilities and their careers through the identification of key themes and research trends. The literature search and review revealed that the articles can be grouped according to five main themes, namely: (1) career inequalities for women (vs. men) with disabilities, (2) barriers to careers for women with disabilities, (3) educational/curriculum intervention to improve career motivations and opportunities for women with disabilities, (4) facilitators/strategies for careers/career development of women with disabilities, and (5) intersectionality of gender, disability, ethnicity, and low socio-economic status (amongst others), and its effects on career development. Collectively, they represent the focal areas of the extant literature on careers for women with disabilities. Further analysis of these themes revealed several gaps in the existing literature, and on this basis, it is suggested that research efforts should be increased in this space, with larger samples conducted in more developing regions, and with more consideration of the context and differentiation of types of disabilities and organisations. This scoping review is thus opportune in laying the foundations for future research examining the career motivations, opportunities, and development of women with disabilities.

## Author contributions

XC is the first contributing author and KH is the second contributing author. The author order reflects contributions to the research and writing of the manuscript. Both authors are accountable for the content of the work, contributed to the article and approved the submitted version.

## Footnotes

1 The bibliometric map was generated using VOSviewer, a text mining software that constructs and visualises co-occurrence networks of important terms extracted from a body of scientific literature.
